# Taking Placebos as Needed to Reduce Appetite: A Randomized Controlled Trial with Ecological Momentary Assessment

**DOI:** 10.3390/bs13030207

**Published:** 2023-02-27

**Authors:** Isabella Unger, Anne Schienle

**Affiliations:** Institute of Psychology, University of Graz, 8010 Graz, Austria

**Keywords:** placebos as needed, compliance, appetite regulation, app-assisted monitoring

## Abstract

Placebos can reduce appetite. However, when placebos are prescribed over a longer period of time, compliance and response rates are not always satisfactory. A new administration approach ‘as needed’ was tested to improve adherence to placebo treatment and its effectiveness. Participants could decide on the time of placebo intake (when their appetite had increased substantially). A randomized controlled trial was conducted over seven days. The participants were allocated to one of two groups: a placebo group (PG; *n* = 41) or a control group with no placebo treatment (CG; *n* = 34). During the intervention, participants used a mobile phone application to rate their daily appetite, mood, and the occurrence of binge-eating episodes in their normal environment. The placebo effect was short-lived; the placebo reduced self-reported appetite only on days 1 and 2 of the trial. The placebo neither influenced mood nor binge-eating frequency. This study found an app-assisted approach with continuous monitoring to be helpful for identifying the temporal course of the placebo response. Future placebo trials should implement this method.

## 1. Introduction

Food-cue exposure can have powerful effects on appetite and eating. Even brief exposure to the sight and smell of food has been shown to increase reported appetite and craving, as well as planned and actual food consumption; this is especially so in individuals high in food cue reactivity (FCR; for a meta-analytical review see [1]). Given the high density of food cues in our environment, it is not surprising that overeating has increased dramatically over the last decades [2]. The results of this include higher levels of overweight and obesity, which increase the risk for certain somatic diseases (e.g., diabetes, heart disease, and some cancers [3]) and mental disorders (e.g., depression [4]). It may be possible to reduce these negative health effects if efficient and easy-to-use treatment strategies to decrease FCR (e.g., pertaining to appetite) were available.

Currently, there are various pharmacological treatments on offer to reduce appetite. However, appetite suppressants often have undesirable side effects, such as gastrointestinal irritation and cardiovascular problems [5]. One therapeutic approach, which does not possess these negative side effects, is placebo treatment. Placebos are inert substances or interventions with no specific effect on the symptoms being treated [6]. The appetite-reducing effects of placebos have been identified in placebo-controlled clinical trials of appetite suppressants [7]. Furthermore, intake of placebos compared to no treatment has been shown to reduce appetite in healthy individuals [8,9] and patients with binge-eating disorder [10].

In studies with healthy weight participants, single-dose placebos to change appetite have been implemented successfully. For example, Potthoff et al. [9] exposed females to pictures depicting combinations of food and non-food items, which were shown once after placebo intake (’appetite suppressant’) and once without placebo intake in a repeated-measures design. The placebo reduced reported appetite as well as the viewing time of the food images. In another study, Hoffmann et al. [8] assigned participants to one of three groups. One group received no placebo (control), whereas the other two received a placebo labeled as an ‘appetite enhancer’ or ‘appetite suppressant.’ Relative to the comparison groups, the ‘appetite suppressant’ reduced reported appetite and increased satiety.

In both of those studies, the placebo was taken only once, which does not mirror the typical administration of appetite suppressants in clinical trials. Thus, in order to evaluate the capacity of a placebo treatment to change FCR, longer intervention intervals are necessary. For instance, Jacobs-Pilipski et al. [10] conducted a four-week-long placebo trial with 451 participants who had been diagnosed with binge-eating disorder (BED). Only 32% of the patients with BED were identified as placebo responders, who reported a reduced frequency of binge-eating episodes (overeating with loss of control). The remaining participants were classified as placebo non-responders. In another longer-term placebo study, Tippens et al. [11] conducted an investigation with adults with obesity (*n* = 114) who were randomized into three groups. Participants in one group were told that they would receive an active ‘weight-loss supplement’ (WLS), in another group participants were told that they would receive a WLS with a 50% chance of it being a placebo, and in the third group, participants received no placebo treatment. After three months, the amount of weight loss did not differ between the three groups. Notably, data from 29% of participants from the WLS group could not be analyzed (e.g., because of nonadherence to the study protocol or reported loss of interest in the study). Furthermore, participants in the ‘weight-loss supplement’ group reported a decline in experienced self-efficacy throughout the study.

In sum, longer-term placebo trials aiming at appetite reduction need to be improved to counteract common problems, such as low placebo response rates, non-adherence, and even drop out. To garner greater placebo effects and increase compliance, a new approach for placebo administration was tested. This novel method of placebo intake ‘as needed’ aimed at helping the participants to regain control over their food intake in critical moments. Participants were instructed to use the ‘medication’ when they felt that their appetite had increased substantially. Along these lines, this study implemented ecological momentary assessment (EMA), which involves the repeated sampling of current experiences and behaviors of individuals in their normal environment [12]. In this present study, along with a seven-day intervention, a mobile phone application (app) was used to monitor participants’ daily appetite, binge eating, and mood. A pop-up function reminded participants each evening to complete their ratings. This app-assisted approach was applied to receive more detailed and continuous ratings/feedback throughout the placebo intervention. Participants were randomly allocated to one of two groups that received either a daily placebo (natural medicine to reduce appetite) or no placebo treatment. It was hypothesized that the placebo group would report less appetite and binge eating as well as improved mood during the seven-day trial compared to the control group. In addition, the placebo group should exhibit greater compliance (i.e., more completed app ratings) relative to the control group.

## 2. Materials and Methods

### 2.1. Participants

A total of 75 participants (63 female) with a mean age of M = 27.41 years (SD = 8.99) and a body mass index (BMI) of M = 25.87 (SD = 4.62) were recruited from a community sample in Austria through advertisements on social media and fliers in supermarkets and restaurants. There was no financial reimbursement for research participants.

**Figure 1:**
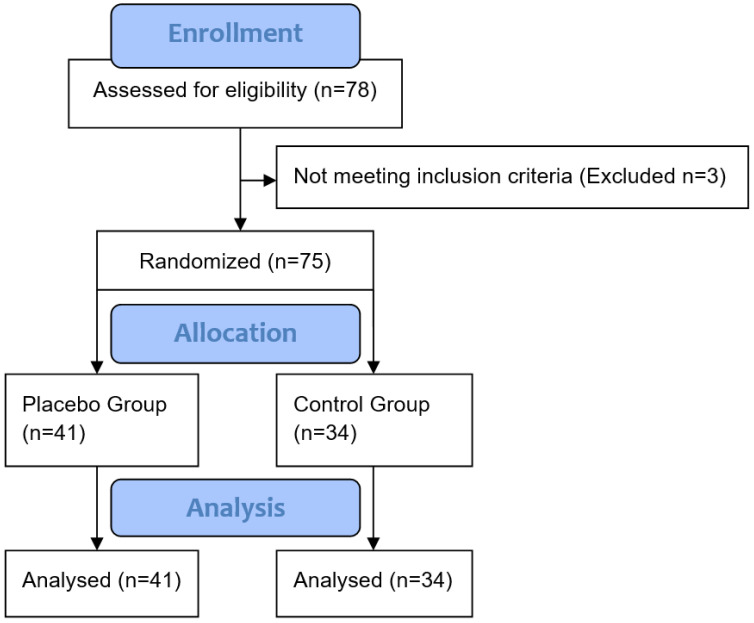
CONSORT flow diagram.

People were invited to participate in this study if they reported high motivation to reduce episodes of overeating (value of 5 or higher; assessed with a ten-point Likert scale ranging from 1 (no motivation) to 10 (strong motivation)). Exclusion criteria were reported diagnoses of eating disorders. Screening for exclusion criteria was conducted via LimeSurvey, an online survey tool. Individuals who reported lifetime and/or current diagnoses of eating disorders, and those who had a BMI < 18.5, were not invited to participate in this study (*n* = 3; see Figure 1). Six participants reported using antidepressants. They were not excluded from the sample because exclusion did not change the results.

In this parallel trial, participants were randomly assigned (with a random number table) by the researchers involved in this study to one of two groups: a control group with no placebo treatment (CG; *n* = 34), and a placebo group (PG; *n* = 41). The two groups did not differ in mean age, BMI, reported food cravings (assessed with the Food Craving Questionnaire—Trait reduced (FCQ-T-r) [13]; Cronbach’s alpha = 0.94), psychological problems (assessed by the Brief Symptom Inventory (BSI) [14]), or participants’ motivation to reduce their overeating (Table 1).

The conducted sensitivity analysis (G*Power [15]) indicated that with a sample size of *n* = 75 and a power of 80% (α  = 0 .05) effects of d > 0.58 can be detected.

### 2.2. Procedure

Written informed consent was obtained from all participants. This study was performed following the recommendations of the declaration of the World Medical Association of Helsinki (revised version, 2000) and the Good Clinical Practice (GCP)—Guidelines (CPMP/ICH/135/95, Final Approval by CPMP 17/07/96). The project was approved by the ethics committee of the University (ethical approval code: GZ. 39/12/63 ex 2019/20).

In the first diagnostic session, participants were asked to come into the lab one by one. All participants completed screening for psychological problems (BSI) and food cravings (FCQ-T-r) and reported their body weight, height, and demographic data (age, education level, and somatic illnesses). The handling of the app was explained to each participant.

Afterwards, the placebo group received water with green food coloring provided in a 30 mL glass bottle with a dropper for oral administration. The food color was calorie-free, sugar-free, and azo-free. The placebo was introduced as herbal medicine (wild garlic: allium ursinum). It was suggested that this substance reduces appetite and overeating. It was further explained that oral application of the fluid (instead of a pill) enables quicker absorption of active agents into the bloodstream and therefore a faster physical response. Additionally, it was mentioned that the herbal medicine had successfully been tested in a clinical trial before. Participants were instructed to take the placebo orally as needed (5 drops) when their appetite had increased substantially. Moreover, participants received a leaflet with information about the placebo. Participants of the CG were instructed to continue their usual eating behavior.

At the end of the 7-day intervention, all participants of the PG rated the perceived effectiveness of the placebo (0: not effective–100: very effective) and returned the placebo bottle to measure the amount of placebo intake (mL). In the CG, participants did not know about the PG and vice versa. All participants were fully debriefed about the study design and the use of the placebo after study completion.

### 2.3. The App

To understand eating behaviors in an ecologically valid way, we used our own in-house custom-programmed app to assess appetite, food cravings, and binge eating on a daily basis in individuals’ natural environment. Data gathering was achieved by combining a PWA (Progressive Web App; Paris, France) and a remote server for storage. The survey was a web page created with HTML, CSS, and JavaScript (using the Vue.js Framework). The anonymous data were sent to a remote server where a Python Flask script handled the data collection and created a CSV file for each participant.

Participants were reminded via a pop-up function from the app every evening to answer questions concerning their mood during the day (valence; from very negative (0) to very positive (100)), appetite (“How hungry were you during the day?” 0: not at all–100: very much), and binge eating (“How many eating attacks did you have today?”).

### 2.4. Statistical Analyses

We first conducted *t*-tests to screen for possible differences between PG and CG in mean age, BMI, psychological problems, reported food cravings, and participants’ motivation to reduce their overeating. Then we performed a compliance analysis comparing the number of completed app ratings between groups (*t*-test). To test the effect of Group (PG, CG) on appetite, mood, and frequency of binge-eating attacks across the one-week trial (mean), we computed *t*-tests. We report Cohen’s d as effect size measure. Alpha was set at 0.05 for statistical significance. Controlling for demographics (age, BMI, gender) did not change the results and was therefore not included in the analyses. To further examine the effects of Group on hunger and mood we conducted mixed-model analyses using the GAMLj package [16] of jamovi (version 2.2.5 [17]). All models included Group (PG, CG) and Day (1–7) as factors with intercept as the random coefficient. The model info was Appetite~1+group+day+group:day+(1|id) and Mood+1+group+day+group:day+(1|id).

Exploratory correlation analyses were conducted for the PG to test the association between the amount of placebo intake, mood, BMI, the motivation to reduce overeating, and the perceived effectiveness of the placebo.

## 3. Results

### 3.1. Compliance

In total, 92% of the participants used the app every day over the one-week course of this study. There was no significant difference concerning the number of completed app ratings between groups across the study interval (t(73) = −0.962, *p* = 0.332; d = −0.22, 95% CI [−0.68, 0.24]; Table 1).

### 3.2. Appetite

Over the one-week trial, there was no difference in reported appetite between groups (t(73) = 1.69, *p* = 0.10; d = 0.39, 95%CI [−0.07, 0.85]; Table 1).

**Figure 2:**
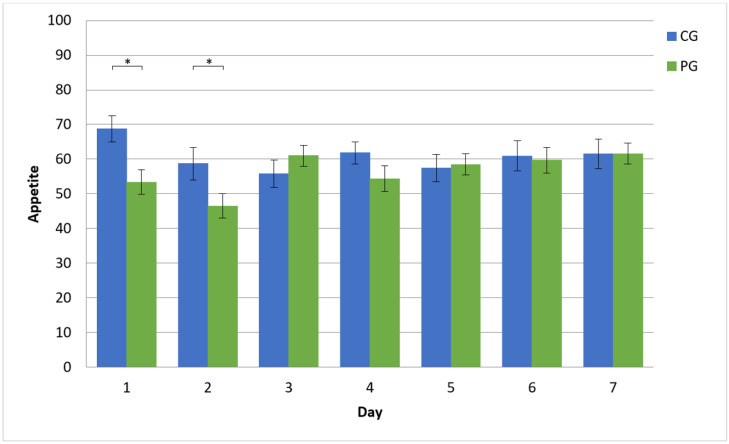
Reported appetite per day and group. * indicates a significant result.

The mixed-model analysis showed no significant main effects of Group (F(1,71.8) = 2.41, *p* = 0.125) or Day (F(6423.3) = 1.73, *p* = 0.113) but did find a significant interaction between Day and Group (F(6423.3) = 2.54, *p* = 0.02; see Figure 2). A simple effect was detected on day 1, indicating a reduced appetite of 15.4 points in the PG compared to the CG (95% CI [−25.60, −5.27], *p* = 0.003) and on day 2, indicating a reduced appetite of 12.2 points in the PG compared to the CG (95% CI [−22.34, −2.16], *p* = 0.017). There were no other significant simple effects for the remaining days (all *p* > 0.144).

Footnote: PG: Placebo Group, CG: Control Group; error bars indicate the standard error of the mean.

### 3.3. Frequency of Binge Eating (FOBE)

There was no difference in the number of binge-eating episodes between groups (t(73) = 1.08, *p* = 0.28; d = 0.25, 95% CI [−0.21, 0.71]; Table 1, Figure S1) across the study interval.

### 3.4. Mood

There was no difference in mood between groups (t(73) = −1.01, *p* = 0.32; d = −0.24, 95% CI [−0.69, 0.23]; Table 1, Figure S1) across the study interval. The mixed model analysis did not show any significant results (Group: F(1,70.8) = 0.970, *p* = 0.328; Day: F(6422.0) = 0.659, *p* = 0.683; Group*Day: F(6422.0) = 0.230, *p* = 0.967).

### 3.5. Ratings of Placebo Effectiveness and Placebo Intake

The following results describe only the placebo group because the control group did not receive a placebo. The perceived effectiveness for the placebo was M = 51.67 (SD = 22.63). Over the study interval, participants took the placebo M = 9.42 times (SD = 9.42; range: 0–48) and the mean amount of placebo intake was 2.4 mL (SD = 2.4, range: 0–12 mL). Three participants who had been assigned to the PG (7%) did not take the placebo.

The amount of placebo intake correlated with mood (r = −0.37, *p* = 0.018) and participants’ BMI (r = 0.38, *p* = 0.013). Participants took the placebo more often when they were in a bad mood and participants with a higher BMI showed higher placebo intake. There was no significant association between the amount of placebo intake and participants’ motivation to reduce overeating (r = 0.18, *p* = 0.268) or the perceived effectiveness of the placebo (r = −0.017; *p* = 0.921).

## 4. Discussion

This study investigated the effects of placebo treatment (‘natural appetite suppressant’) on participants’ appetite, frequency of binge-eating episodes, and mood during a one-week intervention. To enhance placebo effects, the ‘appetite suppressant’ could be taken as needed (in case of an increased desire to eat). Moreover, we used an app-assisted approach to obtain continuous feedback from the participants and to capture the temporal dynamics of the placebo effect.

Up until now, temporal dynamics of the placebo response have rarely been studied. A recent placebo-controlled trial involved repeated measurements. However, the temporal resolution of the assessment was low [18]. Data were collected at baseline, week 6, and week 12 of that study. This study implemented daily assessments and found the placebo effect to be short-lived. The placebo reduced appetite on day 1 and day 2 of this study. Starting with day 3, the two groups did not differ in their appetite ratings. While the positive effects of single-dose placebos for reducing appetite have been reported before [8,9], placebos administered in long-term trials have been less effective. For example, in a study by Tippens et al. [11], a placebo prescribed as an appetite suppressant did not promote weight loss over the three-month study interval. To further understand the temporal dynamics of the placebo effect, more longitudinal placebo studies using a daily app-assisted approach are needed.

Moreover, poor adherence rates or even drop-out are problems related to longitudinal placebo studies on appetite reduction [11,19]. In our study, compliance was very good; a high percentage (92%) of participants completed all of the required app ratings over the trial. One reason might be participants’ high motivation to reduce overeating reported before the trial. Another reason might be the introduction of a placebo ‘as needed.’ Whereas prescribed daily placebo intake might be considered an obligation, placebos administered ‘as needed’ have a voluntary character and aim at helping participants to regain control in challenging times. In line with this intention, placebo intake was higher when participants were in a bad mood and when they had a higher BMI.

However, participants took the placebo on average only nine times during the one-week trial (approximately once a day). Three participants did not take the placebo at all. These participants indicated a low to moderate level of appetite during the trial. Just being able to take an ‘appetite suppressant’ when needed may have already increased their sense of (appetite) control. This hypothesis should be followed up in future research focusing on attitudes and motivations to take (or not take) placebos ’as needed.’

Even though we only recruited participants indicating a high motivation to change their eating behavior (particularly overeating/binge eating), participants in both groups reported a low level of eating attacks over the trial. This demonstrates that continuous monitoring of one’s behavior can serve not only as an assessment method but also as an intervention. Studies on clinically relevant behaviors (e.g., cigarette smoking, alcohol intake, insomnia) found that self-monitoring changes the frequency of the dysfunctional behavior/symptom in the desired direction [20,21,22]. Similar findings have been reported by Latner and Wilson [23], who found a self-monitoring effect on the number of binge-eating episodes. Participants kept continuous records of their food intake, which was sufficient to substantially decrease binge frequency. On that account, using a daily app-assisted approach to obtain continuous feedback from the participants might have worked as an intervention itself in our study.

Several limitations need to be considered when interpreting the present results. First, we tested a sample of predominantly women with overweight but also included individuals with normal weight. Even though including BMI as a covariate did not change our results, greater placebo effects might be expected in individuals with overweight/obesity because of greater motivation for weight loss. Second, we did not monitor eating behavior. Future placebo studies with ‘appetite suppressants’ should additionally assess participants’ food consumption (e.g., calorie intake, amount of food eaten). Third, we used colored water as a placebo instead of placebo pills. Wager and Altlas [24] argue that beneficial treatment experiences in the past as well as positive expectations are needed for meaningful placebo effects. Since pills are used more often as medication than liquids, placebo pills might be more effective. Finally, we administered the placebo with a deceptive suggestion (introduced as herbal medicine). This approach has ethical issues that can be circumvented by using open-label placebos, which have already been successfully applied for various conditions (for a review see [25]).

## 5. Conclusions

Previous studies testing single-dose placebos to change appetite have shown that this approach works in laboratory settings. However, this approach lacks ecological validity. Therefore, this study investigated responses to a placebo that could be taken in the normal environment of the participants ‘as needed.’ The placebo effect on appetite was short-lived (two days). Moreover, the novel ‘as needed’ approach was not associated with a high amount of placebo intake; participants took the placebo approximately once a day. Participants showed a high level of compliance independent of the group assignment. Over 90% of participants used the app every day over the one-week course of this study. This demonstrates that ecological momentary assessment is very useful to monitor the temporal dynamics of placebo responses. Thus, future placebo trials should implement this method.

The current placebo approach needs to be optimized. Subsequent studies on appetite regulation via placebos could use different verbal suggestions (medication instead of natural medicine), and different types of placebos (pills instead of liquids) to boost the placebo effect. Moreover, individuals with a diagnosis of binge-eating disorder or bulimia nervosa might show greater placebo responses. While in the present investigation, the number of reported binge-eating episodes was low, participants with more frequent binge eating might profit more from placebos that can be taken as needed.

## Figures and Tables

**Table 1:** Comparison of the two groups.

	Placebo Group (*n* = 41) M (SD)	Control Group (*n* = 34) M (SD)	t(*p*)	d	95% CI
Age (years)	28.18 (8.97)	25.71 (8.85)	−1.51 (0.14)	−0.35	[−0.81, 0.11]
Body mass index	26.6 (4.6)	25.0 (4.60)	−1.61 (0.11)	0.37	[−0.83, 0.09]
FCQ-T-r score	3.74 (1.11)	4.02 (0.99)	1.17 (0.25)	0.27	[−0.19, 0.73]
BSI score (total)	0.56 (0.45)	0.57 (0.50)	0.10 (0.92)	0.02	[−0.43, 0.48]
MTRO	8.98 (1.54)	9.15 (1.37)	0.54 (0.59)	0.13	[−0.33, 0.58]
Compliance	6.88 (0.46)	6.71 (1.03)	−0.96 (0.33)	−0.22	[−0.68, 0.24]
Appetite	56.6 (12.6)	61.4 (11.5)	1.69 (0.10)	0.39	[−0.07, 0.85]
FOBE	1.39 (1.33)	1.96 (3.03)	1.08 (0.28)	0.25	[−0.21, 0.71]
Mood	65.8 (14.0)	62.5 (14.6)	−1.01 (0.32)	−0.24	[−0.69, 0.23]
PPE	51.67 (22.63)	-	-	-	-
Placebo intake	9.42 (9.42)	-	-	-	-

Footnote: M: mean; SD: standard deviation; FCQ-T-r: Food Craving Questionnaire—Trait reduced; BSI: Brief Symptom Inventory; MTRO: motivation to reduce overeating; Compliance: number of completed app ratings; FOBE: frequency of binge eating; PPE: perceived placebo effectiveness.

## Data Availability

The data presented in this study are openly available in OSF [Open Science Framework] at: https://osf.io/ukt3e/?view_only=83889b1074544d7188ea292e6b657092 (accessed on 23 November 2022).

## Supplementary Materials

The following supporting information can be downloaded at: https://www.mdpi.com/article/10.3390/bs13030207/s1, Figure S1: Reported mood by Day and Group.
