# Supplementary material for: Taking Placebos as Needed to Reduce Appetite: A Randomized Controlled Trial with Ecological Momentary Assessment
Source: Behav Sci (Basel). 2023 Feb 27;13(3):207. doi: 10.3390/bs13030207 (PMC10045637; doi:10.3390/bs13030207)
Supplement: Supplementary file 1 [file behavsci-13-00207-s001.zip › behavsci-2173722-supplementary.pdf]

behavioral sciences

Supplementary

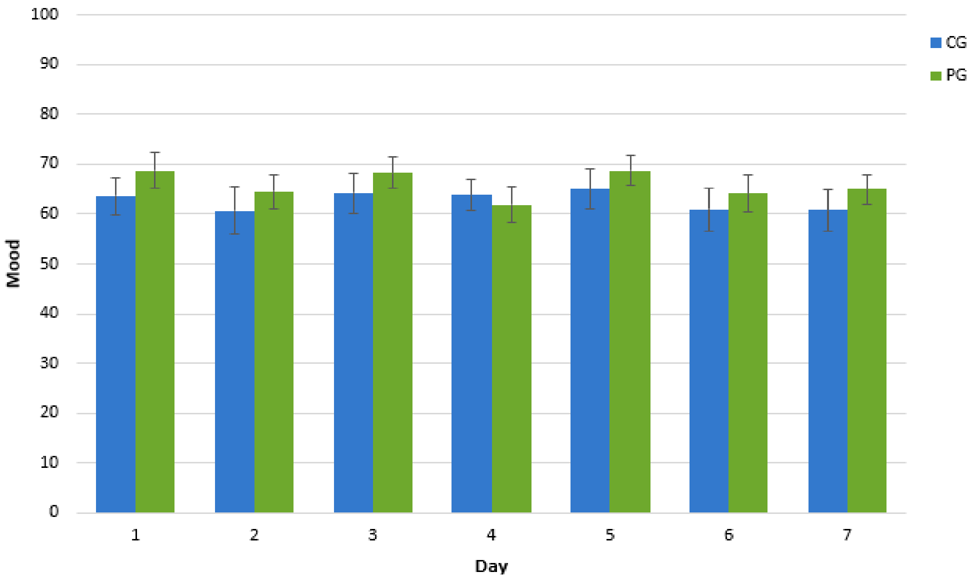

Figure S1. Reported mood by Day and Group. Footnote: PG Placebo Group, CG Control Group; error bars indicate the standard error of the mean
